# Enhanced Circadian Entrainment in Mice and Its Utility under Human Shiftwork Schedules

**DOI:** 10.3390/clockssleep1030032

**Published:** 2019-08-26

**Authors:** Thijs J. Walbeek, Elizabeth M. Harrison, Robert R. Soler, Michael R. Gorman

**Affiliations:** 1 Department of Psychology, University of California San Diego, La Jolla, CA 92093, USA; 2 Center for Circadian Biology, University of California San Diego, La Jolla, CA 92093, USA

**Keywords:** mouse, shiftwork, phase-shift, bifurcation, t-cycle, flexible entrainment, dim light

## Abstract

The circadian system is generally considered to be incapable of adjusting to rapid changes in sleep/work demands. In shiftworkers this leads to chronic circadian disruption and sleep loss, which together predict underperformance at work and negative health consequences. Two distinct experimental protocols have been proposed to increase circadian flexibility in rodents using dim light at night: rhythm bifurcation and T-cycle (i.e., day length) entrainment. Successful translation of such protocols to human shiftworkers could facilitate alignment of internal time with external demands. To assess entrainment flexibility following bifurcation and exposure to T-cycles, mice in Study 1 were repeatedly phase-shifted. Mice from experimental conditions rapidly phase-shifted their activity, while control mice showed expected transient misalignment. In Study 2 and 3, mice followed a several weeks-long intervention designed to model a modified DuPont or Continental shiftwork schedule, respectively. For both schedules, bifurcation and nocturnal dim lighting reduced circadian misalignment. Together, these studies demonstrate proof of concept that mammalian circadian systems can be rendered sufficiently flexible to adapt to multiple, rapidly changing shiftwork schedules. Flexible adaptation to exotic light-dark cycles likely relies on entrainment mechanisms that are distinct from traditional entrainment.

## 1. General Introduction

Many occupational settings, including hospitals, emergency response and transportation, require staff to work at any hour during the day. Working night shifts has been associated with both acute and chronic negative consequences [1]. Acute effects, including increased error rates, are likely a result of both work during the biological night and partial sleep deprivation. With chronic exposure to shiftwork, risks of attrition, and development of physiological and mental health problems increase [2]. The World Health Organization has labeled shiftwork as a probable carcinogen [3], and the American College of Emergency Physicians, to cite just one example, has published guidelines for best practices to mitigate some of its adverse effects [4]. Despite increased awareness of these issues, few effective treatments exist to alleviate these negative consequences. Many attempts to adjust work schedules to optimize circadian health appear unsuccessful, in part due to competing priorities from individuals as well as organizations [5]. As an alternative, a more flexible circadian system could provide a solution to adapt to rapidly changing work and sleep cycles. Enhanced clock resetting has been observed with pharmacological treatments [6,7], or following exposure to constant light [8] or short photoperiods [9,10,11]. While theoretically important, how these findings may be translated for human benefit is unclear.

Accumulating rodent studies have shown that non-invasive environmental manipulations can markedly increase circadian flexibility. First, compared to complete darkness, the mere addition of dim light at night (<0.1 lux) (a) cut in half the time required to re-entrain to acute phase-shifts in the light-dark cycle (LD) [12]; (b) facilitated induction of stable bimodal activity patterns in 24 h light:dark:light:dark cycles (LDLD) (i.e., rhythm bifurcation) [13]; and (c) expanded the range of behavioral entrainment far beyond conventionally understood limits, including 18 and 30 h cycles [14,15]. Second, following rhythm bifurcation under dimly lit nights, hamsters almost immediately adjusted to light:dark (LD) shifts to any phase [16]. In hamsters and mice, the range of entrainment to LDLD cycles was greatly expanded to include 18–30 h zeitgeber periods [14,17,18]. In at least some cases, this enhanced entrainability after bifurcation persisted without the continuation of dim night-time lighting [17]. 

In 2012, our group proposed consideration of such flexible entrainment as a potential strategy for shiftworkers to navigate complex conflicts between work schedules, social obligations, and circadian rhythmicity, with a focus on accommodation of permanent night-shiftwork [19]. In light of the enhanced resetting and T-cycle discoveries since 2012 described above, we see potential for translation to the more common non-permanent shiftwork, where work times may occur at any hour. Thus, here we present three experiments to further characterize rhythm bifurcation and T-cycle entrainment, their underlying mechanisms, and their ability to facilitate adaptation to on-demand schedules. In Study 1 (“Jitter”), we investigated entrainment mechanisms of flexible behavior by probing animals entrained to exotic lighting paradigms with repeated, systematic phase perturbations. In Study 2 (“DuPont”), we used bifurcation to schedule activity according to a common DuPont work routine that alternates blocks of 12 h shifts. Lastly, in Study 3 (“Continental”), we tested whether mice could adapt to a rapidly delaying shift schedule (modified Continental) during weekdays and still be well-entrained to normal conditions on weekends.

## 2. Study Results

### 2.1. Study 1/Jitter—Nature of Entrainment to Bifurcated and Non-24 h Cycles

Previous studies have demonstrated flexible entrainment in lighting regimes generally considered to be far outside the normal range of entrainment, including exposure to both LD (e.g. 13 h of light, 5 h dark; LD13:5 or T18LD) and LDLD (e.g. LDLD5:4:5:4 or T18LDLD) cycles (see methods for explanation of nomenclature) [14,17,18]. Under those cycles that have been carefully characterized, behavioral adaptation reflects bona fide entrainment. Notably, an explanation of simple masking for entrained behavior has been rejected. For example, following both LDLD and extreme T-cycles there is phase control in complete darkness (DD) [14,17,20] and reorganized rhythms of core body temperature [14,21]. In LDLD conditions alone, further evidence includes persistence of biphasic behavior in skeleton cycles [13], enhanced phase-resetting [16,22], entrainment after-effects [20], biphasic melatonin secretion [23], and altered c-Fos and clock gene expression in the Suprachiasmatic nucleus (SCN) [22,24,25]

On the other hand, entrainment to extreme T-cycles lacks the characteristic large phase angle modulation [18] and period after-effects predicted by classical non-parametric entrainment theory [26,27]. This suggests that the enhanced circadian plasticity described above may differ mechanistically from classical entrainment. Study 1/Jitter, therefore, aimed to find evidence of oscillator-driven behavior in T24LDLD, T30LDLD, and T36LDLD. Transient misalignment following a rapid change in the phase of a zeitgeber is indicative of a slow-shifting oscillator. Hence, mice in Study 1/Jitter were exposed to repeated phase-shifts, while the prevalence and magnitude of transients in onsets and offsets of behavior were scored. Any diminution of transients would be interpreted as behavior being controlled by light directly or an extremely rapidly shifting oscillator, rather than by the conventional strong oscillator believed to be the core of mammalian circadian rhythmicity.

### 2.2. Study 1/Jitter—Results

**Figure 1:**
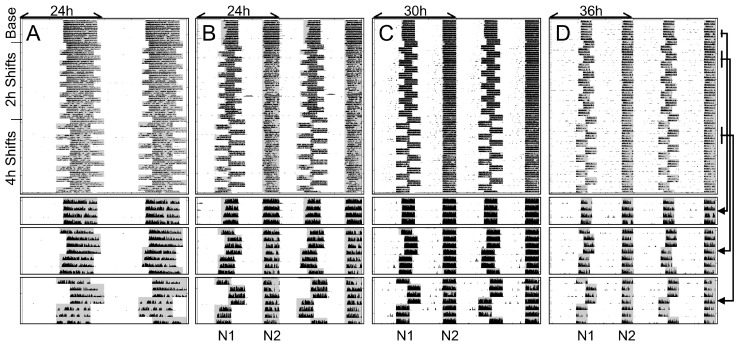
Representative double-plotted actograms of mice in Study 1/Jitter that were exposed to T24. (14:10; (**A**)), T24LDLD (7:5:7:5; (**B**)), T30LDLD (10:5:10:5; (**C**)), or T36LDLD (13:5:13:5; (**D**)), all with dimly illuminated scotophases (<0.1 lux). The second, third, and fourth rows contain blow ups of the baseline, and repeated 2 and 4 h phase-shifts respectively. Wheel running activity is shown in black and scotophases are indicated by gray shading. Each line is scaled from 0–100 revolutions per minute.

Male and female mice were exposed to a standard laboratory photoperiod (14 h light, 10 h dark; LD14:10) as a control or one of three experimental light-dark cycles. All three experimental conditions were first bifurcated in a 24 h light:dark:light:dark (LDLD) cycle. One group remained in that condition (T24LDLD) and the other two groups were subsequently exposed to 30 or 36 h LDLD conditions (T30LDLD and T36LDLD, respectively) as illustrated for representative animals in Figure 1. Each of these animals remained very well-adapted to the light-dark cycles throughout the experiment, as evident from little activity in the light, and no visible free-running components. A measure of equality of division of activity between alternate scotophases in LDLD, the Bifurcation Symmetry Index (BSI; [17]), indicated that mice were well-bifurcated during the first phase of the experiment (0.71 ± 0.08 and 0.56 ± 0.11 for females and males respectively; *t*(10.0) = 1.14; *p* = 0.28). A complementary measure that quantifies behavioral adaptation in T-cycles, Entrainment Quotient (EQ; [17]), showed that mice were likewise well-adapted in T30 baseline (0.96 ± 0.02 and 0.83 ± 0.06 for females and males) and T36 baseline (0.97 ± 0.01 and 0.91 ± 0.05 females and males). Considering data from the two non-24 h conditions, EQ-values were significantly higher in females than males (F(1,28) = 5.93; *p* < 0.05), but did not differ between T30 and T36 (F(1,28) = 1.32; *p* = 0.26), and there was no interaction between T-cycle and sex (F(1,28) = 0.77; *p* = 0.39). 

**Figure 2:**
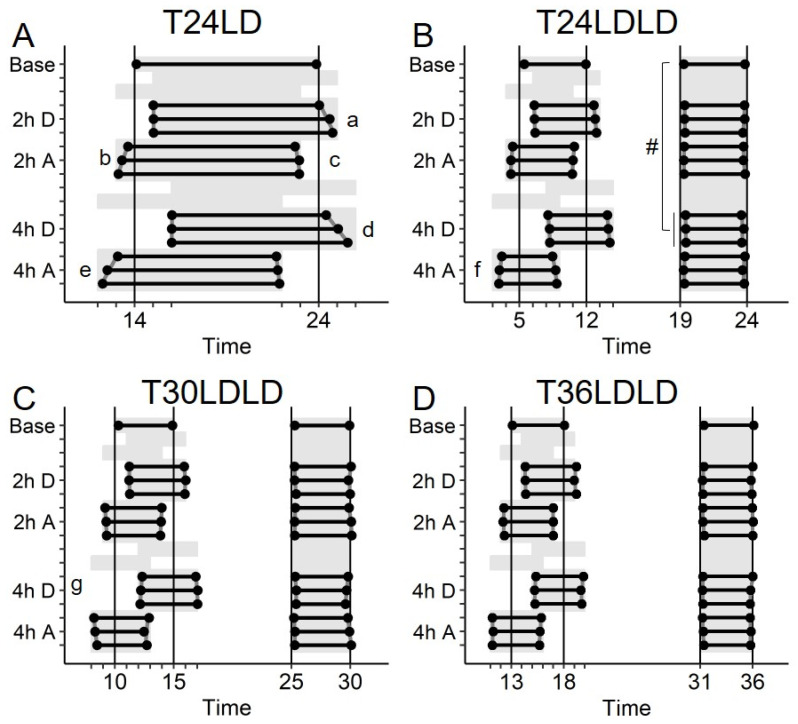
Average activity onsets and offsets in Study 1/Jitter for animals entrained to T24LD (**A**), T24LDLD (**B**), T30LDLD (**C**), and T36LDLD (**D**). Onsets and offsets were averaged across the last 10 days of baseline, and across 5–7 repeats of 2 and 4 h phase-shifts each. Error bars representing SE for onsets and offsets are obscured by data points (all <20 min). Scotophases are indicated by gray background. Significant slopes (min ± SE/day) (i.e., “transients”) are indicated by a lowercase letter: a: 22 ± 4, b: −15 ± 2, c: 7 ± 2, d: 35 ± 6, e: −25 ± 3, f: −7 ± 3, g: 8 ± 3. Positive slopes indicate onsets/offsets occurring later. Significant phase angle differences (min ± SE) compared to baseline are indicated with special characters: #: 9 ± 3. Positive phase angles mean later onsets than baseline. Note the large transients in T24LD (a, b, d, e), but the lack thereof in all other experimental conditions.

After stable entrainment in baseline, mice were exposed to alternating phase delays and phase advances of 2 and 4 h each. In the three LDLD conditions, only every second scotophase was phase-shifted. Figure 2 represents average activity onsets and offsets following delaying and advancing phase-shifts for each of the four lighting paradigms. Following a 2 h phase delay in LD (Figure 2A), onsets were delayed on the first shift, showed no transients over three days, and appeared to be negatively masked by lighting. On the other hand, activity offsets after the first shift were delayed by approximately 1 h, but also showed reliable transients, such that they were delayed by an additional 22 min on subsequent days. The opposite pattern was observed following a 2 h phase advance, where onsets showed a first-day advance of 1.25 h and additional transients of 15 min per day, while activity offsets coincided with the light transition. During the 4 h phase shifts, a comparable pattern of re-entrainment was observed, but delaying and advancing transients were 35 and 25 min per day, respectively. In contrast, in all other conditions transient phase-shifts in onsets and offsets were largely complete on the first day and transients were absent or minimal (7 and 8 min changes, respectively after 4 h advance in T24LDLD (Figure 2B) and 4 h delay in T30LDLD (Figure 2C)). No significant transients were observed in T36LDLD (Figure 2D). If, as theorized elsewhere [13,28], behavior in the LDLD conditions was controlled by strongly-coupled dual oscillators under mediating alternate bouts of activity, we anticipated that phase-shifting one scotophase (N1) may exert spillover effects on the other (N2). Activity onsets in N2, however, did not display any differences in phase angles regardless of phase of N1, except a small relative delay in activity onsets (Δ9 ± 3 min compared to baseline) after 4 h delay in T24LDLD (Figure 2). 

### 2.3. Study 2/DuPont—DuPont Work Schedule with Bifurcation

**Figure 3:**
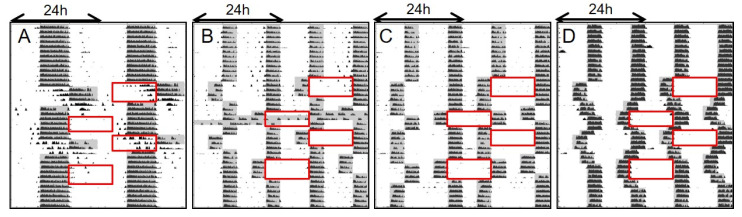
Representative actograms from mice in light:dark (LD) (16:8; (**A**)), and light:dark:light: dark (LDLD) (8:4:8:4) schedules designed to maintain antiphase scotophases (Anti; (**B**)), stable phase angles between scotophases (Phase; (**C**)), or gradual transitions for phase shifts (Step; (**D**)). All scotophases were dimly illuminated (<0.1 lux). For more details on lighting schedules see Section 4.5.2. Superimposed red boxes, single-plotted, represented required work times (Work Blocks 1–4) in a DuPont schedule. In the middle of the experiment a technical failure caused the lights to be off for 48 consecutive hours for group B. Those days were excluded from all analyses, and this did not appear to disrupt behavior after the issue was resolved. Notably, activity in the three LDLD groups appeared to adjust rapidly to the frequent changes in the light schedule.

A DuPont Schedule is a common working schedule in U.S. manufacturing and involves alternating blocks of 3–4 12 h day and night shifts, with non-working recovery days between different shift types. Under normal circumstances, the circadian system simply cannot adapt to the phase adjustments required to work such a schedule [5], but bifurcation in rodents has the potential to greatly enhance readjustments to large phase-shifts [16]. In addition, Study 1/Jitter showed that with bifurcated behavior mice can rapidly adapt to repeated phase-shifts. Therefore, in Study 2/DuPont we tested whether experimental entrainment conditions with bifurcation can facilitate adjustment to a simulated DuPont work schedule. Under LDLD conditions, this necessarily includes adjustments in the phase angle between the twice-daily scotophases, because a symmetric T24LDLD cycle with short photophases (such as LDLD8:4:8:4) does not allow for a long, 12 h work shift. Thus, mice in LD were compared to mice in three alternative scheduling strategies with bifurcation, each approaching the rotation between simulated day and night work shifts differently. All 12 h “work” blocks were scheduled during the photophase, as they would be for diurnal humans. In nocturnal mice, that means that “work” is analogous to scheduling inactivity expected for the subject day. Additionally, successful adaptation to a shiftwork schedule in humans includes efficient sleeping during changing dark intervals. Thus, one goal of this study in a nocturnal rodent model was to produce an entrainment pattern where locomotor activity (a marker of subjective night) was absent from Work Blocks 1–4 (red boxes in Figure 3), but instead occurred robustly in scheduled scotophases (shaded lightly in Figure 3). Combining these two concepts, Percent Activity in the Light can be used as a good indication of overall adaptation [29].

### 2.4. Study 2/DuPont—Results

Prior to evaluating adjustment to a simulated DuPont schedule, mice were entrained conventionally to LD16:8 (control) or bifurcated in LDLD8:4:8:4 and assigned to one of three experimental conditions (*n* = 6/group). Figure 3 depicts a representative actogram from each schedule employed in Study 2/DuPont. The conventionally entrained LD16:8 mice demonstrated predictable shortcomings in adjusting to the changing LD cycle: During the first set of simulated night work shifts (Work Block 1), the representative animal showed significant persistence of subjective night behavior that diminishes over four cycles. The dark periods following these simulated night shifts included robust locomotor activity, but activity stopped well before the end of darkness on the first two nights. There was poor adaptation, moreover, in the adjustment of activity in the first cycle after return to conventional hours, though rest/activity rhythms were largely well-adapted for Work Block 2, which simulates day-work. In Work Block 3 (night-shifts), there was greater intrusion of nocturnal behavior than in Work Block 1. Dark periods after these night shifts had diminished and sporadically-timed activity, indicative of poor adaptation. In Work Block 4, there was good adaptation, as with the previous simulated day-work.

The three experimental groups that incorporated rhythm bifurcation tested alternative approaches to accommodate the work schedule. On “days off”, the first bifurcation group was designed to keep the two 4 h-scotophases in antiphase (Anti), previously hypothesized to be the most stable form of bifurcated entrainment [28]. The second group aimed to keep the phase relationship between the scotophases at LDLD12:4:4:4 or LDLD4:4:12:4 (Phase), to limit the total number of required phase adjustments. For the third group, large phase shifts were replaced by incremental 2 h-steps (Step), as gradual rather than abrupt shifts have been recommended for human rotating shiftworkers [30]. The representative mice in Anti, Phase, and Step (Figure 3B–D, respectively) all appear comparatively well-adapted throughout the experiment and show high alignment of activity with the imposed light-dark cycle during baseline, on simulated work days, and on days off. On average, baseline BSI-values were high for the three LDLD groups and did not differ from each other (0.69 ± 0.07, 0.83 ± 0.02, 0.82 ± 0.04 for Anti, Phase, and Step respectively (F(2,15) = 2.53; *p* = 0.11). 

**Figure 4:**
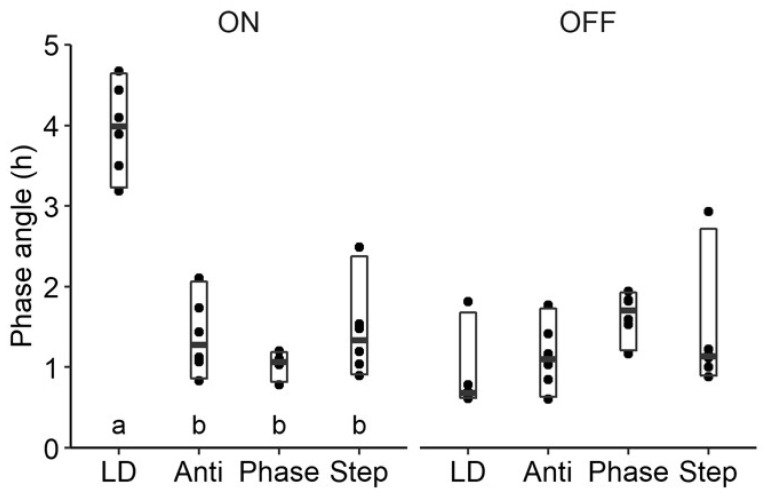
Average absolute phase angles of activity onsets to lights off for mice in Study 2/DuPont on “work days” (left) and “days off” (right). Post hoc group comparisons are indicated with lower-case letters under the groups. Groups that share the same letter were not significantly different from each other. On work days, LDLD groups significantly differed from LD.

As groups were different and continuously changing, simple metrics were used to quantitatively compare the successful adaptation to the DuPont schedule. First, during the 28-day simulated work shift protocol, activity in the light was not different between groups, including LD (0.05 ± 0.02, 0.04 ± 0.01, 0.07 ± 0.02, and 0.12 ± 0.05 for LD, Anti, Phase, and Step respectively, (F(3,20) = 1.3; *p* = 0.30). Additionally, no differences between all groups in average deviations from lights off on days off were found (Figure 4, F(3,20) = 2.50; *p* = 0.08). On work-days, however, LD displayed a large mismatch, with activity starting 4.0 ± 0.2 h away from lights-off (Figure 4), while LDLD groups all showed an average mismatch of less than 1.5 h (1.4 ± 0.2, 1.0 ± 0.1, 1.4 ± 0.2 h for Anti, Phase, and Step respectively; F(3,20) = 49.4; *p* < 0.0001; post hoc group comparisons depicted in Figure 3).

### 2.5. Study 3/Continental—Rotating Work Hours Using T-Cycles

Study 2/DuPont demonstrated that in bifurcation N1 and N2 need neither a stable phase nor phase relationship to maintain entrainment, and that bifurcation facilitated adjustment of wheel activity according to a pre-determined “work schedule”. Study 3/Continental extended these findings by testing if a strategy with T-cycle entrainment can also be used to schedule wheel activity according to human shiftwork paradigms. A T30 (LDLD10:5:10:5) entrainment paradigm allows mice to start activity 6 h later each consecutive day, which could allow a shiftworker to work different shift types on successive days. Therefore, this strategy would be particularly suited for a work schedule involving quickly rotating work times. Here, we used an adapted version of a “Continental” rotating shift schedule, in which real workers rotate between 8 h day, swing, night shifts with days off. Specifically, five 24 h work days (120 h) were replaced with four 30 h cycles (120 h), to allow efficient rotation between different shift types throughout the week. Each 30 h cycle was an LDLD cycle, which allowed two 10 h subjective days (one for work and one personal) and two 5 h subjective nights for sleep. These work-days were alternated with two 24 h weekend days, which allow diurnal days off for the theoretical shiftworker (a social requirement for many). As we hypothesized that bifurcation was needed to maintain entrainment, in the first phase of the study, weekend days were LDLD7:5:7:5, while in the second phase this hypothesis was tested by substituting the LDLD weekends with LD14:10. Mice were exposed to these hybrid T-cycle paradigms with either dimly lit nights following a history of bifurcation—conditions designed to facilitate behavioral adaptation—or with dark nights without the bifurcation-history. The latter condition is typical for laboratory rodents in chronobiological studies. Adaptation was quantified using wavelets and activity in the night. Again, as mice are nocturnal, evidence of good adaptation was absence of activity in the light and presence of activity in the dark.

### 2.6. Study 3/Continental—Results

**Figure 5:**
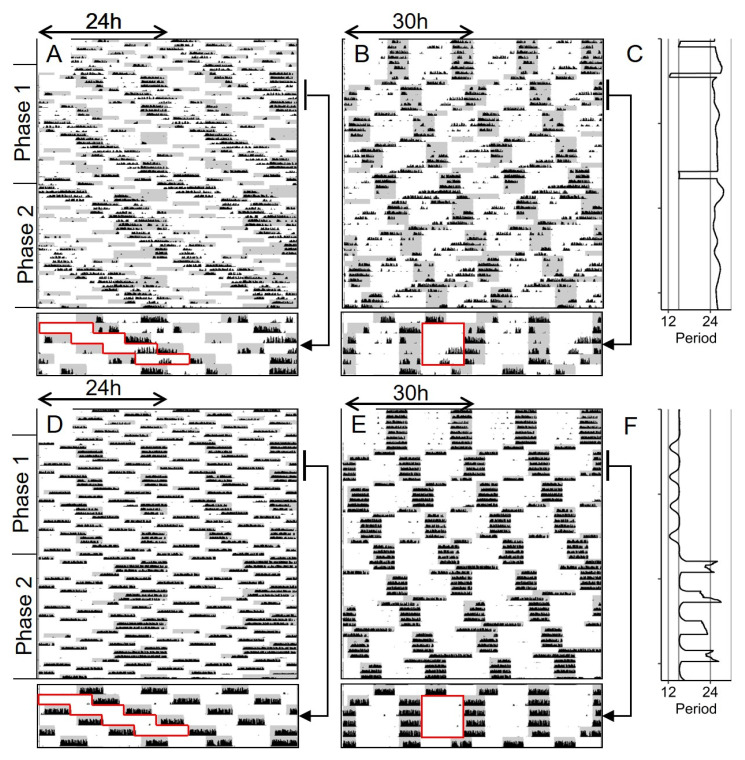
Illustrative double-plotted actograms and wavelet ridge-plots for two animals from Study 3/Continental. Mice were exposed to alternating T24LDLD (7:5:7:5) and T30LDLD (10:5:10:5) cycles in phase 1 and T24LD (14:10) and T30LDLD (10:5:10:5) in phase 2, see Section 4.6. Data for an animal with dark nights is plotted modulo 24 h (**A**) and modulo 30 h (**B**), and data for an animal in the dim night condition (<0.1 lux) are plotted in (**D**,**E**) for modulo 24 and 30 h respectively. Blow-ups at the bottom of each panel represent the first T30 work-week. Red boxes represent a time where four delaying 10 h work shifts could be scheduled. Wavelet ridges for the animals with dark nights and dim nights are plotted in (**C**,**F**) respectively. Vertical gray lines are at 12, 15, 24, and 30 h. Values in the ridge plots for animals in (**F**) alternated rapidly and almost completely between the values matching the changing zeitgebers (12 and 15 in Phase 1 and 15 and 24 in Phase 2), indicating maintenance of entrainment throughout the protocol. Values in (**C**) on the other hand did not follow the period of the light cycle.

Figure 5 shows an example actogram from one animal from each group, with all wheel data plotted modulo 24 h (Figure 5A,D) and modulo 30 h (Figure 5B,E). As anticipated, dim light at night during the stable T-cycles in baseline (Phase 0: top lines in actogram) predicts entrainment, while animals with dark nights mainly fail to entrain. Corroborating the represented individuals, average EQ-values were 0.92 ± 0.03 and 0.67 ± 0.10 for dim and dark, respectively (F(1,20) = 21.2; *p* < 0.001), with females displaying higher EQ-values (0.93 ± 0.02) than males (0.70 ± 0.09; F(1,20) = 17.6; *p* < 0.001).

In Phase 1, all mice were exposed to alternating T30LDLD work weeks and a T24LDLD weekend. The representative dim light animal in Figure 5D,E showed adaptation to both weekend schedules (best visible in modulo 24 h; Figure 5D) and work-week (best visible in modulo 30 h; Figure 5E) as evident from low activity levels in the light and dominant wavelet-periods alternating between 12 and 15 h (Figure 5F). The dark-night animal, on the other hand, predominantly showed free-running rhythms (best visible in modulo 24 h; Figure 5A). Adaptation in Phase 1 of the experiment was highly predictive of behavioral adaptation in Phase 2, where weekend days were un-bifurcated, meaning that free-running animals—a group largely consisting of animals from the Dark condition—continued to free-run, while animals that were behaviorally adapted—mainly from the Dim condition—maintained adaptation.

**Figure 6:**
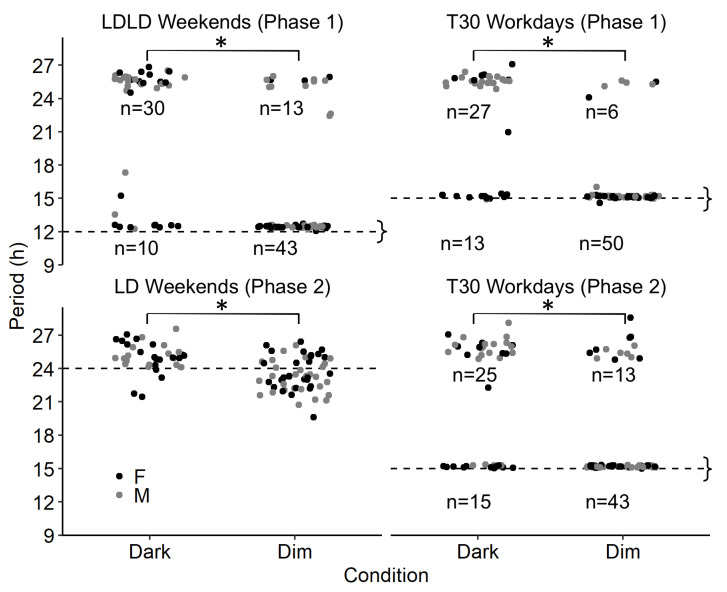
Dominant wheel running periodicity based on wavelet analyses. For each animal, the main period is extracted for each week/weekend separately, therefore each animal is represented in each panel four times. Horizontal dashed line indicates the period of the light-dark cycle, and “}” indicate data points consistent with entrainment. Points deviating from the dotted lines are likely reflective of non-entrained animals, but this is not decisive because some animals could have entrained by frequency demultiplication. The numbers inside the graph indicate the number of data points in each cluster. In T24LDLD weekends and T30 workdays animals with Dim spent more time entrained that animals in Dark. Significant group differences are indicated by *.

Wavelet analysis can evaluate periodicity at any point along a time series, as is shown for the illustrative animals in Figure 5C,F. Wavelets were used to extract the dominant rhythms in wheel running for each 2–4 day block of the schedule by taking a point estimate from the middle of the window. Period estimates generally fell categorically into entrained (i.e., matching the zeitgeber period) and free-running (i.e. >24 h period) values with very few estimates in between (Figure 6). The mice in Dark conditions predominantly display free running rhythms, while mice with Dim nights mainly show rhythms matching the light-cycle (generalized linear mixed effect model; all *p* < 0.05). The only exception is the 24 h LD weekends in Phase 2, where entrained and free-running animals produce overlapping distributions, but the period for animals with dark nights was significantly longer than for animals with dim nights (25.7 ± 0.3 h vs. 23.5 ± 0.2 h; F(1,20) = 38.56; *p* < 0.0001).

**Figure 7:**
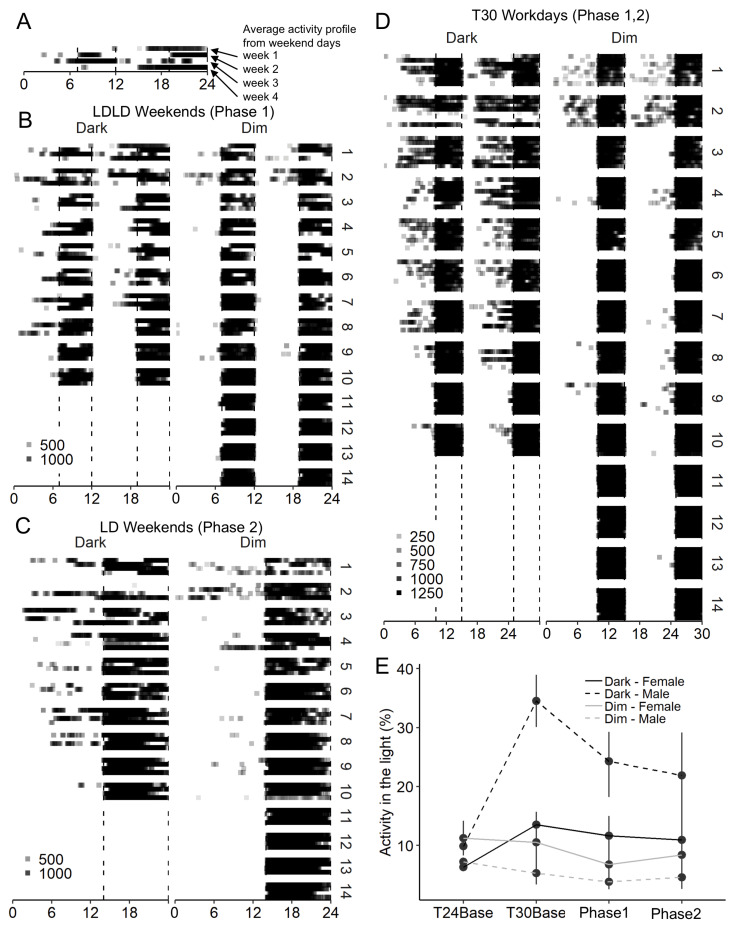
Wheel running activity profiles for each individual in Study 3/Continental from LDLD weekend (**B**), LD weekend (**C**), and T30 weekdays (**D**). A blow-up of the first animals in LDLD weekends/Dark is shown in (**A**). Each block represents four (**A–C**) or eight (**D**) weeks of data from a different animal. Animals were ranked subjectively from worst to best adapted (1–14, listed on the right side of the panels). Within each block, each line represents the averaged activity profiles for two T24 weekend (**A–C**) or four T30 week (**D**) days. Only activity above the daily average is plotted. Intensity of the color represents number of half-wheel revolutions per 6 min bin. (**E**) Average percent activity in the light for each condition represented as mean ± SE throughout the experiment. Activity profiles of animals in Dim were more consistent from week to week and showed less activity in the light compared to animals with Dark nights.

In addition to the wavelet rhythmicity in activity, timing of activity in relation to light-cycles was scored. During T24Baseline, activity in the light did not differ by group or sex (Figure 7E), but during this phase mice with dark nights were still in LD14:10, while mice with dim nights were in LDLD7:5:7:5. In all other phases, activity in the light was greater in Dark than Dim (all *p* < 0.01), and did not differ depending on sex, but sex and condition did interact such that males with dark nights had more activity in the light (all *p* < 0.05).

As in Study 2/DuPont, activity in the light was measured; however, it is sensitive to negative masking. Therefore, we also measured activity in the dark. If a mouse is well-adapted, the full scotophase would be expected to be filled with activity. More importantly, each week- or weekend-day would be expected to have the same respective activity profile. A mouse that is free-running, but masked by the LD cycles, would be expected to have different activity profiles in subsequent weekends depending on the relative phase between internal free-running rhythms and the LD-cycle. Figure 7 represents activity profiles of T24LDLD weekends (Figure 7B), T24LD weekends (Figure 7C), and of all T30 weeks in between (Figure 7D). Animals are sorted based on subjective entrainment. Animals in Dim showed large agreement between weekends, as apparent by near-identical patterns across repeats, while Dark animals showed more of a “checkerboard pattern”, indicative of irregular weekend behavior. 

## 3. Discussion

### 3.1. Summary

Baseline BSI and EQ values corroborate earlier reports that mice are capable of behavioral adaptation to extreme light dark cycles (Study 1–3), that dim light at night facilitates flexible entrainment (Study 3) and that females, on average, adapt to extreme T-cycles better than males (Study 1,3). Furthermore, in Study 1/Jitter, mice in T24LDLD, T30LDLD, and T36LDLD adjusted wheel running activity to a phase-shifted light-dark cycle on the first day, while mice in T24LD show expected transient misalignment in activity onsets and offsets following phase advances and delays respectively. In Study 2/DuPont, bifurcated mice in T24LDLD adjusted their behavior to schedules designed to simulate a DuPont working paradigm better than un-bifurcated animals. Lastly, in Study 3/Continental mice provided dim light at night were capable of adjusting behavior to rapid and frequent changes in T-cycles and this could be used to simulate rotating shiftwork with diurnal days off, while mice with dark nights mainly failed to adjust. Combined, these studies expand the characterization of flexible entrainment associated with bifurcation and T-cycles and strengthen the support for translation to aid shiftworkers with their schedules.

### 3.2. Building Rodent Shiftwork Models

Rodent models of human shiftwork typically assess consequences of circadian disruption similar to that observed in human shiftwork, applying a variety of approaches including forced work during the normal rest phase (e.g., [31]), chronic jetlag (e.g., [32]), and non-entraining T-cycles (e.g., [33]). Few studies compare alternate schedules to minimize circadian disruption [34], and we are aware of no study testing different strategies to achieve a common simulated work schedule in animal models. For Study 2/DuPont and 3/Continental, we therefore developed a strategy where we aimed to evaluate and compare light-dark scheduling practices designed to avoid misalignment. We deployed protocols where adaptation to a predetermined simulated “work schedule” on both days-on and days-off was quantified. This novel approach allows for efficient comparison of alternative scheduling paradigms that are otherwise hard to compare because of the dynamic nature of such experiments. 

Using this method in Study 2/DuPont, three bifurcation-based schedules were compared to an LD cycle for adaptation. As the LD group had the most consistent schedule, with only two adjustments in activity for night-time work-blocks and no further phase-shifts on days off, mice in the control group tended to have better alignment on days off than LDLD animals. Despite having more phase-shifts (as many as 15 in Step Group) and changing photophase duration and phase angles between scotophases, bifurcated mice not only maintained entrainment throughout the experiment, but outperformed un-bifurcated controls on work-days. As no differences were observed between the LDLD groups, invariant phase angles or minimization of the number and magnitude of phase-shifts appear to be unimportant considerations for achieving successful adaptation. Human shiftworkers often break up sleep to accommodate for family and other activity [35]. Given the variety of bifurcation-based schedules that support adaptation to shiftwork schedules, rhythm bifurcation might grant freedom to schedule sleep as desired without sacrificing quality.

While Study 2/DuPont improved alignment to a DuPont schedule, other schedule types are perhaps more common. Study 3/Continental used T-cycles to test adaptation to a delaying rotating shift schedule (modified Continental) with normal weekend days. Indicators of successful adaptation revealed that mice with nocturnal dim light can adjust behavior to match frequently changing periods. As bifurcation on weekends is not necessary to maintain adaptation on work days, hypothetical shiftworkers could remain flexible in how to structure their days off. Together, these experiments show that both bifurcation and T-cycles can be used to schedule rest/activity around a variety of very different simulated real-world shiftwork schedules, without loss of alignment of activity with the light-dark cycle. The scheduling of activities around a given work schedule, despite being one of the few areas of control for the worker, has received little attention historically [36,37]. Proactive scheduling strategies, such as those reported here, might serve as an alternative or even complementary approach. Future studies could directly compare bifurcation and T-cycles and examine the extent to which they might augment one another. Additionally, adaptation may be further improved using timed feeding [38], exercise [39], or other known zeitgebers in addition to light. Furthermore, studies could investigate the consequences for adaptation of extended intermittent periods of normal T24LD entrainment (e.g., long weekends, vacations). Additionally, a Continental shiftwork schedule calls for 8 h delays between subsequent shifts, while T30 only allows for 6 h delays daily. As no qualitative differences between T30LDLD and T36LDLD were found in Study 1/Jitter, we anticipate these findings could be easily extended to a T32 schedule (8 h delays daily), but this should be empirically tested.

The current studies served as a proof of concept that behavioral misalignment is not an inevitable consequence of shiftwork schedules. Behavioral adaptation does not require minimally changing and near 24 h conditions. With bifurcation and dim light, the overall level of adaptation to challenging schedules was greatly increased, even if some animals under such conditions were not ideally adapted. There is little reason to assume that our lighting conditions and schedules were optimal for enhancing circadian flexibility. We have only just begun to evaluate parameters of the lighting environment on entrainment flexibility primarily in T24 LDLD, but see [20]. Factors that predict entrainment to LDLD or T-cycle paradigms include sex, age of the animal, and light parameters like duration and intensity [14,17,20,40,41]. The superior entrainment flexibility in females observed here (in Studies 1 and 3) corroborates previous findings from our lab [14,40], but is seemingly opposite to that reported in humans where males may have higher tolerance than females as reflected in self-reported level of complaints related to sleep or health [42], but see [43]. Mechanisms underlying sex differences in entrainment flexibility in our studies have not yet been investigated. Other individual differences in humans that correlate with shiftwork tolerance include chronotype, age, family structure, and certain personality traits [44]. Awareness of these individual differences, however, has not yet resulted in individualized or actionable solutions to aid shiftworkers. Mouse studies with well-defined shiftwork adaptation endpoints may help distinguish biological factors from social covariates to inform such solutions.

The current report identifies interventions to reduce behavioral misalignment, but the long-term health consequences in any of these extreme lighting paradigms are yet to be investigated. Few formal studies have been published yet, but preliminary observations are encouraging. For example, mice that are successfully entrained in a variety of non-24 h conditions in our lab do not seem to develop an obesity phenotype [14,40], and do not have impaired reproductive function ([45] and unpublished data) as seen in circadian disruption models [2]. Lastly, unlike mice in a simulated jetlag protocol, bifurcated mice do not have deficits in cued memory retrieval in Pavlovian fear conditioning [46]. Even if long term health were not improved, flexible adaptation might alleviate acute effects of shiftwork by improving alertness on the job or the ability to get quality sleep between shifts.

### 3.3. Mechanisms of Behavioral Adaptation

The flexible entrainment to extreme lighting conditions observed here is exceptional and far beyond traditional limits of entrainment [47]. By what mechanism are mice able to adapt their behavior to these highly artificial schedules? A variety of studies with different light cycles and species show converging evidence that an explanation of simple masking can be rejected in favor of a true reorganization of the circadian system [13,14,16,17,20,21,22,23,24,25,48,49,50]. Whereas Study 2/DuPont and 3/Continental took a phenomenological approach to explore overall entrainment under complex photoperiod regimes, Study 1/Jitter was designed to test specific entrainment hypotheses by evaluating oscillator characteristics in bifurcation and T-cycle entrainment in two ways. First, does bifurcation make the system more responsive to light changes? In traditional entrainment, phase-shifts induce transient misalignments between behavior and zeitgeber. These transients are a characteristic of oscillator-driven behavior, and an observable manifestation of the slow rate of adjustment of the central pacemaker after a schedule change. If behavioral adaptation in LDLD relies on similar mechanisms as classical entrainment, transients would be expected in wheel running activity in N1. As in none of the three LDLD conditions did we find transients comparable to those in T24LD, LDLD entrainment renders the system more resettable. The phase-shifts in activity onsets on the first day in response to a phase advance may be a reflection of a resetting action of dark onset and/or incomplete adaptation in this rapidly cycling pattern of advancing and delaying shifts. With respect to transients, changing the T-cycle from 24 to 30 or 36 h did not qualitatively change regulation of behavior. These results complement findings of enhanced phase-resetting in hamsters to simulated time-zone travel [16] and extend the observation to T-cycle entrainment. 

Second, is entrainment in LDLD affected by strong oscillator interactions? If activity in alternating scotophases in non-24 h LDLD were controlled by antiphasic coupled oscillators, as has been proposed in T24LDLD [13,28], phase-shifts in N1 would be expected to also alter activity onsets in N2. The overall independence of activity in N2 from changes in N1 suggests a lack of strong functional interactions between multiple oscillators in these paradigms. The small delay in activity onsets in N2 following a 4 h delay in N1 in T24LDLD is the only spillover effect observed. As in earlier reports with asymmetric 24 h LDLD cycles the dependence of N1 and N2 was in the opposite direction [28], we cannot reject a more parsimonious interpretation that these spillover effects reflect homeostatic mechanisms: with the 4 h N1 delay, there are only 5 h of rest between N1 and N2, which may contribute to the small delay in activity onset in N2. Overall, there is no compelling evidence of interacting oscillators in LDLD nor in T30 and T36.

Combining the observations from all studies, activity rhythms during or following bifurcation and extreme T-cycles appear to be more directly controlled by light than driven by a strong underlying circadian oscillator. At the same time, an explanation of only positive and negative masking of a strong oscillator driving behavior in these conditions has also been rejected by prior evidence [13,14,16,17,20,21,22,23,24,25,48,49,50] as well as by the lack of 24 h rhythmicity in Study 3/Continental (Figure 6). Therefore, we propose an alternative explanation that remains to be empirically tested. While in T24LDLD behavior might be driven by two dissociated circadian oscillators, potentially in subregions of the SCN [48], under extreme T-cycle entrainment, behavior may be uncoupled, wholly or partially, from its normal circadian regulators. Decoupling or partial entrainment of multiple circadian oscillators had been demonstrated rodents in T-cycles [14,51,52,53]. To date, among the best-entrained animals in our studies with dim light and T-cycles, there is no evidence of a free-running circadian oscillator that continues to keep track of 24 h time while behavior is adapted to extreme light cycles. Moreover, upon release in DD, animals rapidly reverted to typical free-running periods with a phase determined by the time of release, rather than at random phases as would be expected from a free-running circadian oscillator or at a specific time which would be predicted by an entrained 24 h-oscillator [14,17,20]. Although our neurobiological investigations have not yet included T-cycles, in bifurcation, clock gene expression in the SCN shows dampened rhythmicity and strong resetting [22]. Recently, another group reported similar enhanced resetting of SCN PER2::LUC rhythms in mice exposed to T-cycles without successful entrainment [54]. Neurobiological studies in progress will test alternative mechanistic hypotheses. Light manipulations, in other contexts, have also led to increased circadian resetting, for example photoperiod manipulations [9,10,11] or short exposure to constant light [8]. Furthermore, both treatment with Vasoactive Intestinal Peptide (VIP) [7] and a serotonin receptor antagonist, NAN-190 [6], have been shown to increase the speed of re-entrainment following a phase shift. Whether dim light-induced enhanced circadian plasticity relies on overlapping mechanisms with any of these remains to be determined.

## 4. Methods

### 4.1. Nomenclature

In non-traditional light-dark paradigms like the ones studied here, standard circadian nomenclature is ill-suited to characterize the complexity of the schedules. In this report, zeitgeber period is noted with a T (e.g., T24 for a 24 h cycle). As prior characterization of bifurcation in T24LDLD demonstrated a 24 h organization [25,28], the LDLD notation is preferred over the logically equivalent T12LD. In T30LDLD and T36LDLD, which are formally equivalent to T15 and T18, on the other hand, no evidence of 30 or 36 h oscillations has yet been found both in current and prior studies [14,17]. Regardless, because of the asymmetric design in phase shifts between N1 and N2 in Study 1/Jitter, the LDLD notation is used there. For consistency, the same notation is used in Study 3/Continental. 

### 4.2. Housing and Lighting

During all experiments, mice (C57BL/6) were singly housed in shoebox cages (28 × 18 × 17 cm) and provided with a running wheel (13 cm diameter). Cages were placed in light-tight, ventilated chambers that fit up to 16 cages each. Chambers were equipped with fluorescent lamps that provided 327 ± 162 lux at the cage level during photophases, and dim green LEDs (555 ± 23 nm) that could provide illuminance no greater than 0.1 lux (irradiance of 3.90 × 10^−5^ W·m^−2^) in the brightest parts of the cage during the scotophase. Use of dim lights is indicated in experimental details and denoted as LD_ark_ or LD_im_. Room temperature was continuously monitored and regulated at 22 ± 2 °C. Food (Mouse Diet 5015; Purina, St. Louis, MO) and water were provided ad libitum and wheel-running activity was recorded continuously (VitalView Version 4.2, Mini-mitter, Bend OR) as the number of half wheel revolutions per 6 min bins. Before the experiments, mice were co-housed with same-sex siblings (3–4 per cage) in the colony in LD_ark_14:10 without a wheel. At the end of the experiments, animals were humanely euthanized. All experiments received approval of the Institutional Animal Care and Use Committee, University of California, San Diego before being conducted and followed all university guidelines. 

### 4.3. Quantitative Assessments of Entrainment and Analyses

To objectively quantify behavioral adaptation to extreme light-dark cycles, two metrics were deployed. First, for evaluating bifurcation in 24 h LDLD cycles, the Bifurcation Symmetry Index (BSI) quantifies symmetry in the distribution of activity between alternate scotophases. For each 24 h-cycle, the scotophase (N1 and N2) with the lesser number of activity counts is determined. The sum of all activity counts in that dark interval is then divided by the total activity across 24 h (Total24) and multiplied by 2 (Daily score = 2 * min (N1,N2)/Total24). Daily symmetry values are then averaged across 10 days to yield a BSI score. This provides an objective measure ranging from 0 (all activity consolidated in one of the two nights) to 1 (completely symmetrical activity). Any light-time activity—which in large amount indicates poor entrainment—also lowers BSI. 

Similarly, for non-24 h light-dark cycles, an Entrainment Quotient (EQ) was calculated to evaluate behavioral adaptation to the zeitgeber period. Using 10 24 h-days of wheel-running data, Lomb Scargle periodograms were created to evaluate rhythmicity at any period. Peak periodogram power (PPP) at the period of the LD cycle (e.g., 18 h in T18) is an indicator of entrained behavior (PPP_entrained_), while peak periodogram power in the circadian range (i.e., 23–26 h) is an indicator of non-entrained, or free-running components (PPP_circadian_). EQ is calculated by dividing PPP_entrained_ by the sum of both (EQ = PPP_entrained_/(PPP_entrained_ + PPP_circadian_). EQ gives an objective evaluation of fully entrained (EQ approaches 1) or free-running (EQ approaches 0) behavior. Free-running rhythms combined with negative and positive masking (induced by light and dark, respectively) will likely result in intermediate EQ values.

Study 3/Continental used fast-changing T-cycles, which do not allow for sufficiently long stable behavior needed for EQ determinations. Therefore, wavelet analyses were used to evaluate the period of the dominant frequency at each point in time. Clocklab was used to perform wavelet analyses using the default settings (Start: 2 h; End: 30 h; Step: 0.1 h; Cycles: 8; Sigma; 2, Bin: 6 min). Ridge data—consisting of the highest power period at each point in time—were extracted from the full frequency spectrum. For each mouse, the dominant period for each 120 h work-week and 48 h weekend block was extracted by taking the average ridge value from the 6 h surrounding the midpoint of the window. Entrainment was defined as dominant period within T-cycle period ±1 h.

Activity onsets and offsets were eye-fitted in ClockLab. Activity was recorded using VitalView. Periodogram-analyses and the creation of actograms were performed using ClockLab version 2.72, while wavelet analyses were done in ClockLab version 6.0.26. All other statistical analyses and plotting were done using R.

### 4.4. Study 1/Jitter

#### 4.4.1. Stable Entrainment

Group-housed male and female mice from the colony (5–11 weeks of age) were moved to single housing with running wheels during the first 2 h of the light-phase, and divided into four groups. The first group (LD; *n* = 10, half female) was exposed to 14 h of bright light and 10 h of dimly illuminated relative darkness (LD_im_14:10). The other three groups were exposed to LD_im_LD_im_7:5:7:5 (Table 1). For the transition to LDLD, animals were placed in darkness (with dim light) for 5 h (N1) at the end of the 2 h transition window then 7 h of photophase, followed by another dark phase (N2) that started at time of original lights off, but was truncated. After 2 weeks of LDLD, the three groups were separated: one group (Bifurcation, *n* = 14, half female) remained on LD_im_LD_im_7:5:7:5; a second group (T30, *n* = 16, half female) was exposed to LD_im_LD_im_10:5:10:5; and a third group (T36, *n* = 16, half female) was exposed to LD_im_LD_im_13:5:13:5. Both light-schedule transitions were implemented by extending the photophases while always keeping every scotophase 5 h.

#### 4.4.2. Repeated Phase Shifts 

After 2 weeks, mice were stably entrained. Animals in group LD were then exposed to a 1 h phase delay for three days. This shift was not analyzed but was performed to render future advances and delays symmetric with respect to the initial entraining conditions. From here, mice were exposed to repeating cycles of 2 h phase-shift every 3 days alternating between phase advances and phase delays. After 7 repeats, there was one transitional 3 h phase delay, followed by another seven repeats of alternating 4 h phase advances and 4 h phase delays, all occurring every 3 days.

The groups with LDLD light cycles (Bifurcation, T30 and T36) were exposed to a similar pattern of repeating 2 h, then 4 h, phase-shifts. Importantly, in these groups only N1 was phase-shifted, while N2 maintained a stable phase, causing the two scotophases to not be antiphasic anymore but rather asymmetrical. As each cycle is longer than 24 h, we had fewer iterations of these shifts so that they were performed over the same 12 week interval as the shifts in LD. 

#### 4.4.3. Exclusion of Non-Entrained Animals

For each of the three LDLD conditions, only the eight most well-entrained—defined by BSI and EQ values from the last 10 days of baseline—were included in the analyses. Results and conclusions did not depend on decisions for exclusion: although not reported, qualitative results did not change with post-hoc analyses using different criteria. 

#### 4.4.4. Onset and Offsets/Activity in the Light

For every day in the experiment, activity onsets and offsets were determined for every scotophase and expressed in relation to the light transitions to calculate phase angles. In the LDLD cycles this was done for N1 and N2 separately. Using mixed effect linear regression, prevalence and magnitude of transients—non-zero slopes of phase angles across 3 days—were determined in N1. Averaged phase angles for the four experimental phases in N2 were compared to baseline. Any significant slopes or changes in phase angle smaller than the temporal resolution of the measurement (6 min recording bins) were not reported. Total activity during the light was calculated for each 24, 30, or 36 h cycles and averaged across days for each phase.

### 4.5. Study 2/DuPont 

In Study 2/DuPont, mice in both standard and bifurcated conditions were exposed to a variant of a Dupont work schedule, requiring four, large-magnitude phase adjustments across the course of four weeks. 

#### 4.5.1. Stable Entrainment

Group-housed male mice aged 4–5 weeks were divided into four groups (*n* = 6/group). For two weeks, the first group was exposed to LD_im_16:8, and the other three were exposed to LD_im_20:4. Animals were then moved to individual cages equipped with running wheels. For the LD_im_16:8 group, this occurred shortly before lights off. For the other three groups, wheels were introduced at the beginning of the new, second scotophase in an LD_im_LD_im_8:4:8:4 cycle to facilitate uniform bifurcation across animals. Animals then remained in baseline conditions (LD_im_16:8 or LD_im_LD_im_8:4:8:4) for 4 weeks to allow for stable entrainment before the experimental phase commenced.

#### 4.5.2. Experimental Phase

After 4 weeks, all groups were exposed to a simulated DuPont shift-schedule. A DuPont schedule is a commonly-used shiftwork schedule in U.S. manufacturing and consists of alternating blocks of 3–4 12 h day (e.g., 8 a.m.–8 p.m.) and night (8 p.m.–8 a.m.) shifts with days off in between. To accommodate night shifts, the LD group was phase delayed by 8 h prior to the first night shift and phase advanced back to the original phase after. The LD group’s schedule was based upon self-reported behavior of typical shiftworkers (e.g., staying up all day to work a first night shift, and then sleeping after) [55]. The three bifurcated groups each had their own scheduling strategy to adjust their activity-rest schedule to work shifts. The first group maintained scotophases in antiphase wherever possible and only changed the phase angle when needed to accommodate 12 h work shifts (Anti). The second group maintained a stable phase relationship whenever possible, even on days off (i.e., LDLD12:4:4:4; Phase). The third group minimized the magnitude of individual phase-shifts by adjusting gradually in 2 h shifts rather than 4 h as in the other groups (Step).

#### 4.5.3. Quantification of Adaptation

To quantify adaptation, activity in the light and activity onsets were determined. Onsets were separately calculated for work days (defined as the first onset after a work schedule until the last onset after the last day of each work-block) and days-off. Activity in the light was calculated over the entire 28 day protocol starting with the day of Work-Block 1.

### 4.6. Study 3/Continental

A separate cohort of male and female mice (7–11 weeks old) was assigned to one of two groups upon transition from the colony to singly housing with wheels. The first group (Dark, *n* = 10, half male) was maintained on 14 more days of LD_ark_, while the second group (Dim, *n* = 14, half male) was exposed to 14 days of LD_im_LD_im_ to facilitate flexible entrainment. From here on, both groups were exposed to identical bright-light schedules. The dim light illumination, however, remained as it was before in each respective group. Following the 24 h cycles, both groups were exposed to six cycles of LDLD 10:5:10:5 (Phase 0), followed by four repeats of four 30 h “work days” (equivalent in length to five 24 h days), and two bifurcated 24 h “weekend days” (Phase 1). The six T30LDLD cycles of baseline, combined with the first workweek provided 10 full cycles of stable T30 to quantify entrainment using EQ-values. In Phase 2, four additional weeks of T30 work-weeks with T24 weekends followed. The weekends, however, were now unimodal LD cycles rather than LDLD (Table 2).

## 5. Conclusions

Together, the present studies demonstrate that entrainment in these exotic interventions is regulated by mechanisms that are distinct from classical entrainment. Furthermore, adaptation is compatible with multiple large perturbations as often occurs in real-world shiftwork settings. Therefore, bifurcation and T-cycle paradigms may have translational potential to aid shiftworkers in coping with complex schedules.

## Figures and Tables

**Table 1:** Lighting schedule for Study 1/Jitter.

	Phase 0: Stable Entrainment		Phase 1: 2 h-Phase Shifts	Phase 2: 4 h-Phase Shifts
LD (*n* = 10)	28 cycles: LD_im_ 14:10		7 × 6 cycles: LD_im_ 14:10	7 × 6 cycles: LD_im_ 14:10
Bifurcation (*n* = 14)	28 cycles: LD_im_LD_im_ 7:5:7:5		7 × 6 cycles: LD_im_LD_im_ 8:5:6:5/6:5:8:5	7 × 6 cycles: LD_im_LD_im_ 9:5:5:5/5:5:9:5
T30 (*n* = 16)	14 cycles:LD_im_LD_im_ 7:5:7:5	11 cycles:LD_im_LD_im_ 10:5:10:5	6 × 6 cycles: LD_im_LD_im_ 11:5:9:5/9:5:11:5	6 × 6 cycles: LD_im_LD_im_ 12:5:8:5/8:5:12:5
T36 (*n* = 16)	14 cycles: LD_im_LD_im_ 7:5:7:5	10 cycles: LD_im_LD_im_ 13:5:13:5	5 × 6 cycles: LD_im_LD_im_ 14:5:12:5/12:5:14:5	5 × 6 cycles: LD_im_LD_im_ 15:5:11:5/11:5:15:5

**Table 2:** Study 3/Continental light schedules.

	Phase 0: Stable Entrainment		Phase 1: Bifurcated Weekends	Phase 2: Non-Bifurcated Weekend
Dark (*n* = 10)	14 cycles: LD_ark_ 14:10	Six cycles: LD_ark_LD_ark_ 10:5:10:5	Four repeats of: 4× LD_ark_LD_ark_ 10:5:10:5, 2× LD_ark_LD_ark_ 7:5:7:5	Four repeats of: 4× LD_ark_LD_ark_ 10:5:10:5, 2× LD_ark_ 14:10
Dim (*n* = 14)	14 cycles: LD_im_LD_im_ 7:5:7:5	Six cycles: LD_im_LD_im_ 10:5:10:5	Four repeats of: 4× LD_im_LD_im_ 10:5:10:5, 2× LD_im_LD_im_ 7:5:7:5	Four repeats of: 4× LD_im_LD_im_ 10:5:10:5, 2× LD_im_ 14:10

## Abbreviations

LD	Light Dark
LDLD	Light Dark Light Dark
BSI	Bifurcation Symmetry Index
EQ	Entrainment Quotient
N1	Night 1
N2	Night 2
SCN	Suprachiasmatic nucleus
